# Multiple Episodic Evolution Events in V1R Receptor Genes of East-African Cichlids

**DOI:** 10.1093/gbe/evu086

**Published:** 2014-05-06

**Authors:** Masato Nikaido, Tomoki Ota, Tadashi Hirata, Hikoyu Suzuki, Yoko Satta, Mitsuto Aibara, Semvua I. Mzighani, Christian Sturmbauer, Kimiko Hagino-Yamagishi, Norihiro Okada

**Affiliations:** ^1^Department of Biological Sciences, Graduate School of Bioscience and Biotechnology, Tokyo Institute of Technology, Yokohama, Japan; ^2^Department of Biosystems Science, School of Advanced Sciences, The Graduate University for Advanced Studies (Sokendai), Hayama, Japan; ^3^Foundation for Advancement of International Science, Tsukuba, Japan; ^4^Tanzania Fisheries Research Institute, Dar es Salaam, Tanzania; ^5^Department of Zoology, Karl-Franzens-University Graz, Austria; ^6^Department of Dementia and Higher Brain Function, Integrated Neuroscience Research Project, Tokyo Metropolitan Institute of Medical Science, Tokyo, Japan; ^7^Department of Life Sciences, National Cheng Kung University, Tainan, Taiwan

**Keywords:** accelerated evolution, chemo-detection, d*N*/d*S*

## Abstract

Fish use olfaction to detect a variety of nonvolatile chemical signals, and thus, this sense is key to survival and communication. However, the contribution of the olfactory sense to social—especially reproductive—interactions in cichlids is still controversial. To obtain insights into this issue, we investigated the genes encoding V1Rs—possible candidates for reproductive pheromone receptors—among East-African cichlids. Interestingly, we found an excess of nonsynonymous over synonymous substitutions in four of six V1R genes in multiple cichlid lineages. First, we found that highly dimorphic *V1R2* allele groups were shared among the cichlids inhabiting all East-African Great Lakes emerged through the episodic accumulation of the nonsynonymous substitutions prior to the radiation of the Lake Tanganyika species flock. We further detected such episodic events in *V1R1* of the tribe Tropheini, and in *V1R3* and *V1R6* of the tribe Trematocarini. The excess of nonsynonymous substitutions in these examples were indicated as d*N*/d*S* > 1, which were all statistically significant by Fisher’s exact test. Furthermore, we speculate that the amino acid changes in these episodic events are likely functional switch because they occurred in the putative ligand-binding pocket. Our finding of the occurrence of multiple episodic events and the unexpected gene diversity in one unique gene family is suggestive of the contribution of the V1R to the species diversification and the social interaction in cichlids.

## Introduction

Most fish rely on olfaction for feeding and to mediate many social behaviors, including reproduction, migration, kin recognition, and aggression (Sorensen and Stacey 2004). Olfactory cues and the relevant behaviors have been identified in various fishes; these cues include migratory pheromone in sea lamprey (Sorensen et al. 2005), priming pheromone in goldfish (Dulka et al. 1987), and male-attracting pheromone in masu salmon (Yambe et al. 2006). Because chemical signaling may be crucial under water, each fish has developed a highly sophisticated olfactory system for communication. However, the importance of olfactory communication in species-rich African cichlids has not been elucidated.

Each of the East-African Great Lakes, namely Tanganyika (LT), Malawi (LM), and Victoria (LV), harbors several hundred cichlid species, which are both ecologically and morphologically diverse (Turner et al. 2001; Kocher 2004). Phylogenetic and geographical studies suggest that the cichlids of each lake have arisen independently from a small number of ancestral species followed by extensive diversification in an evolutionarily short period. As such, the cichlids provide a textbook example of adaptive radiation, which may help to unravel the genetic mechanisms that underlie animal diversification. Kocher (2004) categorized the cichlid speciation events into three stages: 1) choice of habitat, 2) morphological diversification due to trophic status, and 3) diversification of male color pattern followed by that of mate recognition. Thus, visual cues are of primary importance for the last stage of cichlid radiation. Indeed, several molecular studies (Terai et al. 2002, 2006; Sugawara et al. 2005; Seehausen et al. 2008) have shown adaptive and divergent evolution of opsin genes, suggesting the importance of visual cues for cichlid radiation. Although biologists have paid less attention to olfaction than to vision, several recent studies propose that olfaction also contributes substantially to cichlid reproductive communication (Crapon de Caprona 1980; Miranda et al. 2005; Cole and Stacey 2006; Barata et al. 2008). Furthermore, other recent studies found that mating preference in cichlids may be based partly on olfactory cues (Plenderleith et al. 2005; Verzijden and ten Cate 2007). These reports imply that olfaction plays a greater role in cichlid social communication than previously thought.

V1R is a recently characterized fish olfactory receptor gene family (also known as ORA) (Pfister and Rodriguez 2005; Saraiva and Korsching 2007). The repertoire of the fish V1R gene family is small (i.e., six genes in most species), and it is rigidly maintained in distantly related fish species including cichlids (Saraiva and Korsching 2007; Grus and Zhang 2009; Ota et al. 2012; Nikaido et al. 2013a). Genes orthologous to teleost fish V1Rs have also been found in lamprey, elephant shark, and frog, indicating that these genes have been maintained for an extremely long period during the evolution of aquatic vertebrates (>500 Myr; Grus and Zhang 2009). Furthermore, each of these *V1R* orthologs is highly conserved at the nucleotide and amino acid sequence levels among distantly related rockfish species (Johansson and Banks 2010) as well as among salmonid species (Johnson and Banks 2011). These studies suggest the operation of strong purifying selection, implying that V1Rs are functionally indispensable for the survival of aquatic vertebrates. These attributes are in striking contrast to other olfactory receptor gene families (e.g., OR, V2R, and TAAR), which have large and highly variable gene repertoires owing to extensive lineage-specific gene expansions (Niimura and Nei 2005; Hashiguchi et al. 2008; Nei et al. 2008; Nikaido et al. 2013b). Although the ligands of fish V1Rs are currently unknown, their unique features imply that they bind a small set of evolutionarily conserved chemicals, such as “hormonal pheromones” (Saraiva and Korsching 2007; Johansson and Banks 2010). Hormonal pheromones are metabolites of steroids or prostaglandins that ensure the synchronization of gamete maturation and/or spawning interactions (Stacey 2003).

To examine the possible involvement of V1Rs in the social interaction of cichlids, we determined and compared the sequences of all six V1R genes of more than 30 cichlids species inhabiting the three East-African Great Lakes and surrounding rivers. Surprisingly, we found that the V1R genes are highly diversified in sequence among some lineages of cichlids because of multiple episodic accelerations of amino acid substitutions. We here describe the evolution of six V1R genes in East-African cichlids and discuss the potential of social or reproductive communication in cichlids that use the highly diversified V1R receptors.

## Materials and Methods

### Fish and DNA Samples

The fish species used in this study are listed in supplementary table S1*A* and *B*, Supplementary Material online. LV specimens were collected during an exploration that was led by the Okada laboratory from 2005 to 2007. Locations of the sampling sites are summarized in supplementary table S1*B*, Supplementary Material online. Other cichlids were caught in the wild or purchased from a commercial source. Tissues from fresh-caught fish were fixed in 100% ethanol and stored at 4 °C. DNA was extracted using the DNeasy Tissue kit (QIAGEN).

### Polymerase Chain Reaction and DNA Sequencing

Primers used for V1R gene characterization are listed in supplementary table S2*A*, Supplementary Material online. Polymerase chain reaction (PCR) and sequencing were performed as described (Terai et al. 2002). Briefly, to determine the allelic phase of heterozygotes (the coding region and flanking sequences), DNA fragments were cloned into pGEM-T (Promega) and separated into two sequences. These sequences were subsequently used for both phylogenetic and population genetic analyses. For sliding-window analyses of *V1R2* of *Haplochromis sauvagei*, long PCR was used to amplify DNA fragments, which included coding sequences and both upstream and downstream flanking sequences (∼5 kb). Sequences determined in this study are available at GenBank (accession numbers AB670359–AB670682, AB671354–AB671436, and AB704543–AB704712).

### Phylogenetic and Population Genetic Analyses

DNA sequences were edited using GENETYX-Windows version 5. MEGA 5 software (Tamura et al. 2011) was used to construct neighbor joining trees to estimate the ancestral sequences and to calculate genetic distance and divergence time. The MODELTEST version 3.7 program (Posada and Crandall 1998) was used to determine the model of DNA-sequence evolution that best fit our data sets. To run MODELTEST, PAUP* 4.0b10 (Swofford 2003) was used. The PHYML program (Guindon et al. 2005) was used to construct the ML tree based on the MODELTEST model. The haplotype network tree was constructed using TCS (Clement et al. 2000). The *π*, Tajima’s *D* values, and degree of linkage disequilibrium (*D*′) were calculated using DnaSP 5 (Librado and Rozas 2009). The sequence logo of V1Rs was generated using a web-based program, Weblogo 3.3., available at http://weblogo.threeplusone.com (last accessed May 1, 2014) (Crooks et al. 2004).

### Genomic Southern Hybridization

Genomic DNA (10 µg) of *H. chilotes* (heterozygote of clades I and II of *V1R2*) and *Tropheus duboisi* (homozygote of clade I of *V1R1*) was digested with EcoR*I*, Hind*III*, Pst*I*, or Bgl*I*, and then subjected to 0.8% agarose gel electrophoresis. DNA was transferred to GeneScreen Plus charged nylon membrane (PerkinElmer USA) using standard protocols. Approximately 1-kb fragment, which included the entire coding V1R sequence, was labeled with digoxigenin (DIG) using a PCR DIG probe-synthesis kit (Roche). Hybridization was carried out in a solution containing 25% formamide, 7% SDS, 5× SSC, 0.1% *N*-lauroylsarcosine, 50 mM phosphate buffer (pH 7.0), and 2% blocking reagent (Roche) at 42 °C overnight. Washes were performed in 0.1× SSC containing 0.1% SDS at 65 °C. Hybridized probes were detected using alkaline phosphatase-conjugated anti-DIG Fab fragment and CDP-Star (Roche), and they were visualized using Kodak Image Station 2000R (Kodak).

### In Situ Hybridization

Olfactory organs of LV cichlid (*H. sauvagei*, homozygote of V9 allele of *V1R2*) and Tropheini cichlid (*T. moorii*, homozygote of clade II allele of *V1R1*) were sectioned horizontally at 7 μm. Sections were hybridized with a DIG-labeled cRNA probe, which was synthesized using a DIG RNA labeling mix (Roche). Sections were fixed in 4% paraformaldehyde for 10 min. Hybridization was performed at 55 °C overnight as described (Kashiwagi et al. 2006). Alkaline phosphatase-conjugated anti-DIG Fab fragment and the chromogen NBT-BCIP (Roche) were used to visualize the positive signals.

## Results

### Two Major *V1R2* Allele Groups Are Found in LV Cichlids

We recently characterized six V1R genes in LV cichlids using a *H. chilotes* BAC library (Ota et al. 2012). To evaluate the variability of each V1R gene, we first analyzed genetic divergence by comparing V1R gene sequences from 17 species of LV cichlids (one individual from each species) (supplementary fig. S1*A*, Supplementary Material online). The cichlids used in this analysis are summarized in supplementary table S1*A*, Supplementary Material online. We observed an exceptionally high genetic distance in *V1R2* (1.05%), whereas *V1R1*, *V1R3*, *V1R4*, and *V1R5* showed very low genetic distances (0.01–0.05%). The latter results are consistent with analyses of other nuclear genes (Samonte et al. 2007). We also found relatively high sequence divergence in *V1R6* (0.54%), but this analysis will be presented elsewhere as the conclusions drawn from *V1R6* of the LV cichlids differ somewhat from those drawn from *V1R2*. We thus first focused our analysis on *V1R2*, attempting to identify the genetic mechanisms responsible for generating such exceptionally high nucleotide diversity within closely related LV cichlids.

**Fig. 1.—:**
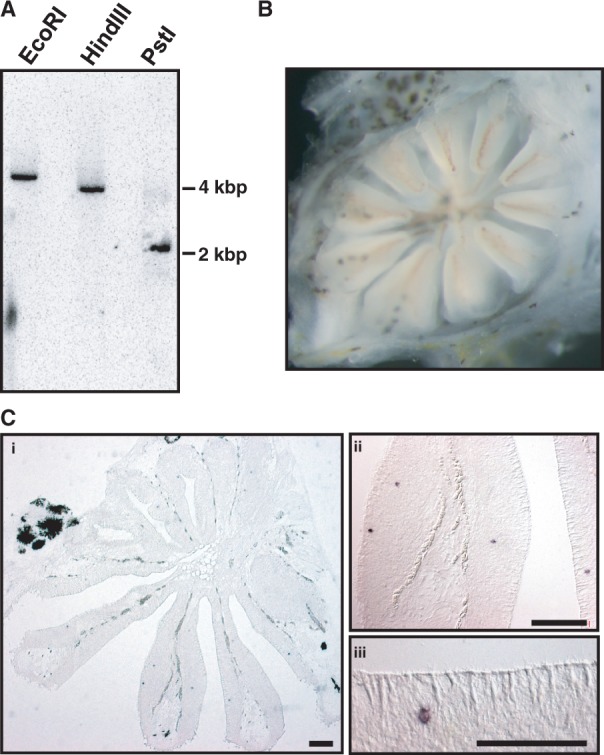
*V1R2* copy number and mRNA expression within the olfactory epithelium. (*A*) Southern blot analysis of genomic DNA isolated from *Haplochromis chilotes* (heterozygote of V1 and V9) using a *V1R2* probe. DNA was digested with EcoR*I*, Hind*III*, or Pst*I* before electrophoresis. Size markers are indicated to the right. Photographs from two independent experiments were combined. (*B*) The photo of the olfactory organ (called “olfactory rosette”) of the adult individual of *H. sauvagei*. (*C*) Three magnifications of the thin sections of the olfactory rosette are shown. Horizontal sections (7 µm) were hybridized with a DIG-labeled cRNA probe to evaluate *V1R2* (V9 allele) expression. Scale bars indicate 100 µm.

The highly divergent *V1R2* sequences prompted us to examine whether multiple copies of *V1R2* are present in the cichlid genome. We carried out Southern blot analysis of genomic DNA isolated from one individual of *H. chilotes*, which possesses highly divergent *V1R2* sequences, revealing a single hybridizing band in each of the digests (fig. 1*A*). Furthermore, when the PCR products of *V1R2* from a single individual were sequenced, we never obtained more than two different sequences. Thus, *V1R2* is likely a single locus (see also Discussion). To evaluate the expression of *V1R2* at the cellular level, we performed in situ hybridization on sections of the olfactory rosette, which is the olfactory sensory organ (fig. 1*B*). Within the olfactory epithelium, the *V1R2* mRNA was detected in sparse cells of the sensory neurons (fig. 1*C*).

**Fig. 2.—:**
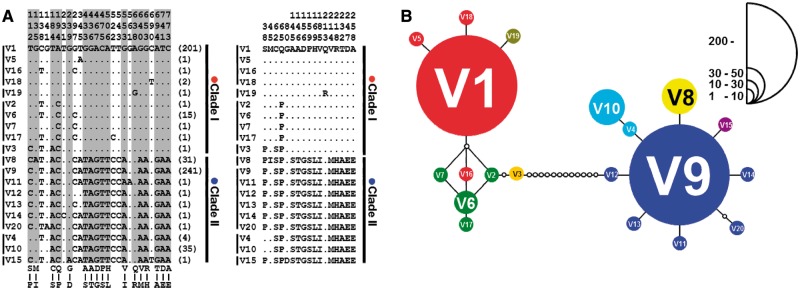
Dimorphic diversity of *V1R2* in LV cichlids. (*A*) Nucleotide (left) and amino acid (right) sequence alignment. Dots indicate sites that are identical to the top sequence (V1 allele). The number of times each sequence was identified is shown in parentheses. Alleles with identical amino acid sequences are grouped with a vertical line. (*B*) Network tree of the *V1R2* alleles. Each circle represents an allele described in panel (*A*). The size of the circle is proportional to the allele frequency (scale is indicated). Alleles with identical amino acid sequences are shown in the same color. Hypothetical alleles, which serve as connectors between observed alleles, are represented by white circles.

We then investigated intraspecific variations of *V1R2* sequences within 271 LV cichlids (supplementary table S1*B*, Supplementary Material online, summarizes the species, genotype, and location of each individual). The alleles were divided into two large groups (clade I and clade II) based on the sites of variable nucleotides. Each clade consists of ten different alleles. These 20 alleles include 2 major-dominant alleles (V1 and V9), 3 relatively major alleles (V6, V8, and V10), and 15 minor alleles (fig. 2*A* and supplementary table S1*B*, Supplementary Material online). At the amino acid level, these alleles encoded eight different sequences. Figure 2*B* shows a haplotype network tree of *V1R2* alleles from LV cichlids. Clade I is dominated by the V1 allele, and clade II is dominated by V9. The frequencies of *V1R2* alleles among species or populations of LV cichlids are categorized as two cases. In one case, the alleles appear to be fixed or almost fixed in several species or populations; in the other case, alleles of clades I and II exist in a polymorphic state within species or populations (supplementary fig. S1*B*, Supplementary Material online). Although we comprehensively investigated the ecological and morphological characteristics between two groups of cichlids, each of which is fixed by the allele with the same *V1R2* clade, no obvious commonality was found among them. Furthermore, we examined deviations from Hardy–Weinberg equilibrium in the populations of *H. sauvagei*, which shows highly dimorphic allele frequency (64 individuals, supplementary table S1*B*, Supplementary Material online), and did not found a significant deviation from Hardy–Weinberg equilibrium.

### Highly Dimorphic *V1R2* Allele Groups Are Shared Among LV, LM, and LT Cichlids

It is unusual that two highly divergent allele groups exist within the populations of LV cichlids, which were diverged just 15,000 years ago (Johnson et al. 1996). Accordingly, we analyzed an additional 1.5 kb upstream and downstream of the *V1R2*-coding region. We performed sliding-window analysis of *π* and Tajima’s *D* for 16 *H. sauvagei* individuals. Higher *π* values and significantly positive Tajima’s *D* were detected within *V1R2*-coding sequences and noncoding regions downstream of the gene (supplementary fig. S2*A* and *B*, Supplementary Material online). Furthermore, most of the coding-region mutations were tightly linked to mutations within the 3′-noncoding region, implying that recombination was rare across this 2-kb region during evolution. Actually, the significant degree of linkage disequilibrium was detected between the coding region and the 3′-region (*D*′ = 0.881, *P* < 0.001 by two-tailed Fisher’s exact test; supplementary fig. S2*B*, Supplementary Material online). As the noncoding regions are inherently free from selective pressure associated with amino acid substitutions, unexpectedly high nucleotide diversity within this 2-kb region may have resulted from the retention of older alleles (see below).

We next estimated the divergence time of clade I and clade II alleles. Silent substitutions within *V1R2*-coding and 3′-noncoding sequences from 17 LV cichlids (same as the supplementary fig. 1*A*, Supplementary Material online) were used for this analysis. The silent substitution rate was calculated from the 5′-flanking sequences of *V1R2* from 35 East-African cichlid species. We assumed that LV and LM cichlids diverged 2 Ma and that haplochromines and LT species flocks diverged 8 Ma (Kocher 2004). The calculated rate was 1.25 × 10^−^^9^ to 1.44 × 10^–9^ substitutions per site per year, which is comparable to the rate calculated in another study (1.1 × 10^–9^ substitutions per site per year) (Won et al. 2005). Thus, the divergence time between clade I and clade II alleles was estimated to be 8.3–9.6 Ma, which predates the radiation of the LT species flock (Kocher 2004).

**Fig. 3.—:**
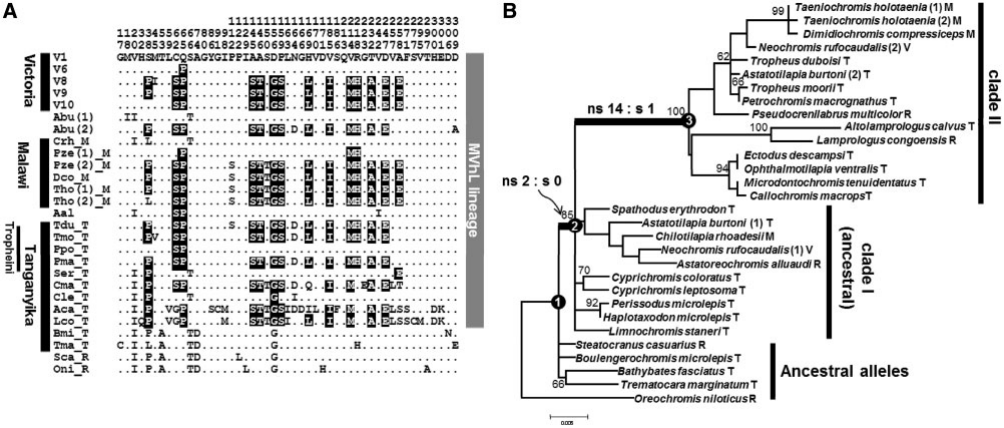
Dimorphic diversity of *V1R2* among East-African cichlids. (*A*) Alignment of VIR2 sequences from East-African cichlids. Only variable sites are shown in the alignment. Dots indicate residues that are identical to the top sequence (V1 allele from LV). Diagnostic amino acids to the V9 allele in the MVhL cichlids (Takahashi et al. 2001) are highlighted in black. Abbreviations of species names are listed in supplementary table S1*A*, Supplementary Material online. (*B*) ML tree of *V1R2* and detection of positive selection. The ML tree of *V1R2* alleles of cichlids constructed by PHYML under the HKY+I+G model. The possible recombinant sequences between clades I and II were excluded from the analysis to reconstruct an accurate genealogy. Bootstrap values more than 60 are indicated above each node. Scale bar indicates the number of amino acid substitutions per site. The phase of each heterozygous *V1R2* allele in the individuals is indicated in parentheses. The letters after the species names (V, M, T, and R) indicate the location of the species as LV, LM, LT, and rivers, respectively. The topology is consistent with that expected from the evolution of *V1R2* in cichlid in that each of the clades (I and II) is monophyletic and they split before the divergence of the ancestor of MVhL lineages. The number of nonsynonymous and synonymous substitutions on the branch is indicated by ns:s.

To further investigate the evolutionary history of *V1R2*, we compared *V1R2* sequences of cichlids from LT, LM, and nearby rivers. Figure 3*A* shows an alignment of these *V1R2* amino acid sequences. The amino acid replacements that identify clade II are highlighted. We rarely observed heterozygotes within LM and LT cichlids, which implies that, at the species level, *V1R2* is almost fixed to either clade I or II in these lakes. Indeed, we confirmed this fixation by analyzing multiple individuals from several species of LT cichlids (eight individuals of *Cyprichromis coloratus*, *C. leptosoma*, and *Paracyprichromis brieni*). The most intriguing result of this analysis was that both clade I and II alleles were shared by cichlids of all East-African lakes and rivers. Such a mosaic distribution of these alleles in all three East-African lakes is not consistent with cichlid phylogeny, in which each of the LV, LM, and tribe Tropheini (LT) forms a monophyletic group (Kocher 2004). To examine this issue at the nucleotide level, we constructed a maximum-likelihood (ML) tree. The ML tree clearly indicates that the *V1R2* alleles split into clades I and II with essentially maximal bootstrap probability (fig. 3*B*). Furthermore, each of the clades contains cichlids from LT, LM, and LV. This analysis indicates that the ancestral *V1R2* alleles split into clades I and II in the ancestor of the MVhL lineage (haplochromines and modern LT cichlids, which include Lamprologini; Takahashi et al. 2001, see fig. 3*A*).

Given that ancestral East-African cichlids (*Oreochromis niloticus* and *Steatocranus casuarius*; Terai et al. 2003) and also those in West-Africa, South America, and Madagascar have clade I-like sequences (supplementary fig. S3, Supplementary Material online), clade I alleles are likely ancestral. Within the ML tree, the branch-connecting clades I and II were unexpectedly longer than the other branches (fig. 3*B*, shown by a thick branch), implying a high rate of nucleotide substitutions during the emergence of clade II. Ancestral sequences were estimated using the ML method (MEGA). An excess of nonsynonymous to synonymous substitutions (14:1) was detected on the branch leading to clade II. The ratio of divergence at nonsynonymous and synonymous site (d*N*/d*S* ratio) of the branch was estimated to be 6.24. The d*N*/d*S* ratio was significantly larger than 1 according to the Fisher’s exact test (Zhang et al. 1997) (*P* = 0.03; table 1). When we investigated the branch connecting node 1 and node 3, such signature became more significant. This analysis suggests that the operation of positive selection (or relaxation of purifying selection) accelerated amino acid substitutions in *V1R2*.

**Table 1:** Signature of the Excess of Nonsynonymous over Synonymous Substitutions

Gene	Branch	ns	s	Significance
*V1R1*	c.a. of Tropheini	11	0	0.024*
*V1R2*	c.a. of MVhL lineage	14	1	0.030*
		16	1	0.016*
*V1R3*	From c.a. of Trematocarini to *Trematocara marginatum*	19	2	0.022*
*V1R6*	From c.a. of Ectodini to			
	*Cyathopharynx furcifer*	14	1	0.138 ^a^
	*Callochromis macrops*	11	0	0.122 ^a^
	From c.a. of Trematocarini to			
	*Trematocara unimaculatum*	10	0	0.032*
	*Trematocara marginatum*	21	1	0.005**

Note.—c.a., common ancestor, ns, nonsynonymous substitution; s, synonymous substitution.

^a^Not significant by Fisher’s exact test.

*Significant at 5% level.

**Significant at 1% level.

To examine whether the sequences of clade I and clade II of *V1R2* are paralogs or orthologs, we constructed neighbor-joining trees of nearby 5′- and 3′-sequences flanking *V1R2*-coding region (∼1 kb) separately for a broad range of cichlid species (supplementary fig. S4, Supplementary Material online). Namely, if the sequences of clades I and II are derived from paralogous loci, the flanking sequences are also supposed to be separated according to the clade I or II. Importantly, in the phylogenetic tree of the 5′-flanking sequences, the clades I and II were not separated into two clades, suggesting that these sequences are not paralogous but orthologous (supplementary fig. S4*A*, Supplementary Material online). The 3′-flanking sequences, however, tend to be separated according to the clade I or II that could be explained by the rarity of recombination between two alleles across the coding region and the 3′-flanking region (supplementary fig. S4*B*, Supplementary Material online).

### Similar Dimorphism in *V1R1*

**Fig. 4.—:**
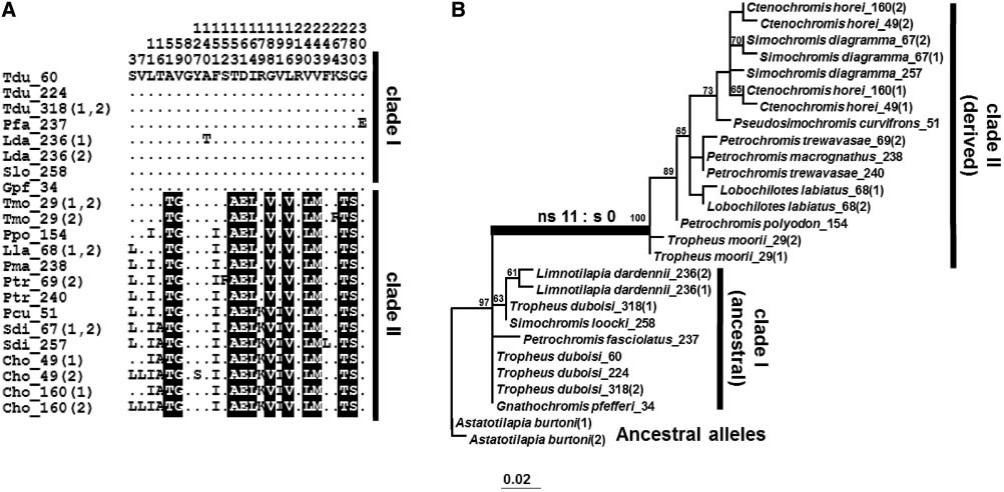
Dimorphic diversity of *V1R1* in the tribe Tropheini. (*A*) Alignment of amino acid sequences of V1R1 in 17 cichlids of tribe Tropheini (12 species). Only variable sites are shown. A dot indicates a site identical to the top sequence (*Tropheus duboisi*). The phase of each heterozygous *V1R1* allele in the individuals is indicated in parentheses. The heterozygous at the synonymous sites are indicated as (1, 2). The sequences were divided into two major allele groups (clade I and clade II) based on the variable sites. Abbreviations of species names are listed in supplementary table S1*A*, Supplementary Material online. The ID number of each cichlid sample is indicated after the species name. (*B*) ML tree of *V1R1* in cichlid tribe Tropheini. The ML tree was constructed under the GTR+I model, based on an alignment of 25 sequences of *V1R1* in cichlid tribe Tropheini. The tree was rooted with *Astatotilapia burtoni*. Bootstrap values more than 60 are indicated above each node. The number of nonsynonymous and synonymous substitutions on the branch is indicated by ns:s.

The finding of the highly dimorphic diversity in *V1R2* prompted us to further investigate other V1R genes in cichlids (supplementary fig. S5*A–E*, Supplementary Material online). Interestingly, we found that the branch was obviously long in the tribe Tropheini in the phylogenetic tree of *V1R1* sequences of 35 East-African cichlids (supplementary fig. S5*A*, Supplementary Material online, shown by a thick branch). To further investigate the pattern of nucleotide substitution in *V1R1* of Tropheini, we analyzed additional species belonging to this tribe. Figure 4*A* shows the alignment of the deduced amino acid sequences of Tropheini *V1R1*. A total of 17 individuals (12 species) were compared in this alignment. Similar to *V1R2*, the *V1R1* alleles were separated into two major groups, clades I and II, between which 11 diagnostic substitutions were observed (fig. 4*B*). *V1R1* is apparently fixed to either the ancestral or derived allele group at the species level in Tropheini (*T. duboisi*: 6 individuals and *T. polli*: 15 individuals). An excess of nonsynonymous to synonymous substitutions (11:0) was also detected in *V1R1* on the branch leading to clade II. We then performed the statistical test to examine whether the d*N/*d*S* ratio is more than 1 in *V1R1*. This test accepted the d*N*/d*S* > 1 at the 5% level (*P* = 0.038; table 1), implying the operation of positive selection (or relaxation of purifying selection).

### Additional Signatures for Episodic Evolutions on *V1R3* and *V1R6*

Next, we tested for an excess of nonsynonymous substitutions in *V1R3* and *V1R6*, in which obviously long branches were observed in the phylogenetic tree (supplementary fig. S5*B* and *E*, Supplementary Material online). For *V1R3*, an excess of nonsynonymous substitutions was observed on the branch connecting the basal node of the tribe Trematocarini to *Trematocara marginatum* (see supplementary fig. S5*B*, Supplementary Material online). Furthermore, an excess of nonsynonymous substitutions was observed again on the branches of *V1R6* for the two species of the tribe *Trematocarini* (supplementary fig. S5*E*, Supplementary Material online). The d*N*/d*S* > 1 for these examples were all statistically significant by Fisher’s exact test. Although it was not statistically significant, the number of nonsynonymous substitutions was apparently higher on the branch connecting the basal node of the tribe Ectodini to the *Callochromis macrops* (supplementary fig. S5*E*, Supplementary Material online). We did not find such accelerated amino acid substitutions in *V1R4* and *V1R5* (supplementary fig. S5*C* and *D*, Supplementary Material online).

### Sequence LOGO and the Position of Amino Acid Changes

**Fig. 5.—:**
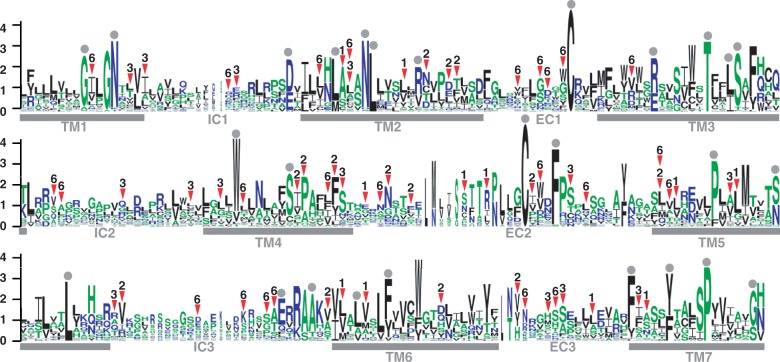
The position of amino acid substitutions accumulated in the episodic events. Conserved sequence motifs of teleost V1R proteins are displayed as a sequence logo. The relative frequency of amino acids at given sites is reflected by the height of the single-letter amino acid code. The trans-membrane (TM), extracellular (EC), and intracellular (IC) regions are indicated by bold gray bars. The gray circles above the residues indicate the amino acid sites putatively responsible for ligand binding (not for ligand selectivity) in V1Rs and the other GPCRs, which were predicted in several studies (Katada et al. 2005; Niimura and Nei 2005; Pfister and Rodriguez 2005). The arrowheads and the numbers 1 and 2 indicate that the amino acid replacements accumulated in the given V1R genes.

We investigated the locations of amino acid substitutions accumulated during periods of rapid evolution. The sequence logo (Crooks et al. 2004) shows the degree of conservation in all teleost V1R genes (fig. 5). The sequence comparison of various G-protein-coupled receptors (GPCRs) suggested that a total of 27 amino acid positions (gray circles in fig. 5) were essential to the basic function of teleost V1Rs (Pfister and Rodriguez 2005; Saraiva and Korsching 2007). We plotted all amino acid replacements that occurred during the episodic events on *V1R1*, *V1R2*, *V1R3*, and *V1R6* (shown by arrowheads). In the logo, we found that a substantial number of amino acids were changed at EC2, TM5, and TM6, which form the ligand-binding pocket and thus contribute to the selectivity of GPCRs for various chemicals (Katada et al. 2005). In contrast, no amino acid substitutions occurred at the 27 functionally essential positions.

## Discussion

### Clade I and Clade II Alleles of *V1R2* Are Orthologous or Paralogous?

The mosaic distribution of the two *V1R2* allele groups across the East-African cichlids suggests that they emerged prior to the radiation of the LT species flock and were subsequently sorted into LT, LM, and LV (see later Discussion). However, it is possible that the gene duplication occurred at the *V1R2* locus in the ancestral population of East-African cichlids, and only one of the two genes were detected by PCR, namely, clades I and II are paralogous. However, such possibility is apparently eliminated by several lines of data shown in our study: 1) A single hybridizing band was observed in the Southern blot analysis using the genomic DNA of an individual with heterozygote for the clades I and II (fig. 1*A*). 2) We never obtained more than two different sequences in one individual. 3) The 5′-flanking sequence was not separated into two groups regardless of clade I or clade II (supplementary fig. 4, Supplementary Material online). 4) The allele frequencies of *V1R2* did not show significant deviations from Hardy–Weinberg equilibrium in the LV cichlid populations. 5) We found only one putatively orthologous *V1R2* locus in the draft genome of the five East-African cichlids, which are available in the database (web site: BouillaBase.org). Thus, we concluded that the sequences of clades I and II of *V1R2* are derived from a single locus. Other than *V1R2*, the single locus of *V1R1* in Tropheini was also inferred by the database search and the genomic Southern blot analysis for *T. duboisi* (supplementary fig. S6, Supplementary Material online).

### Functional Change or Loss in V1Rs After Episodic Event?

Although the episodic accumulation of amino acid substitutions in V1Rs likely implies functional changes, we also need to examine the possibility of functional loss. For example, because cichlids are believed to be vision-oriented fish, it is possible that the environmental constraint became relaxed on olfactory receptor genes in some cichlids, which may have led to the fixation of nonfunctional alleles. Given that a substantial number of amino acids were changed at EC2, TM5, and TM6 in the putative ligand-binding pocket (fig. 5), the V1Rs encoded by different allele groups are likely to be functionally distinct. Furthermore, no amino acid substitution at the 27 functionally essential positions and the absence of large deletions or frameshift mutations implies that these genes are functional. Thus, the accumulation of amino acid substitutions in four V1R genes during episodic events likely indicates the “functional change” rather than the functional loss.

In addition, we detected the expression of *V1R1* and *V1R2* mRNAs by in situ hybridization on sections of the olfactory rosette of *T. moorii* (supplementary fig. S6, Supplementary Material online) and *H. sauvagei* (fig. 1*C*), respectively. Given that both individuals were homozygous for the clade II allele, the data provide the additional evidence that *V1R1* and *V1R2* are functional even after the episodic events.

### Retention of the Dimorphic Alleles in *V1R1* and *V1R2*

**Fig. 6.—:**
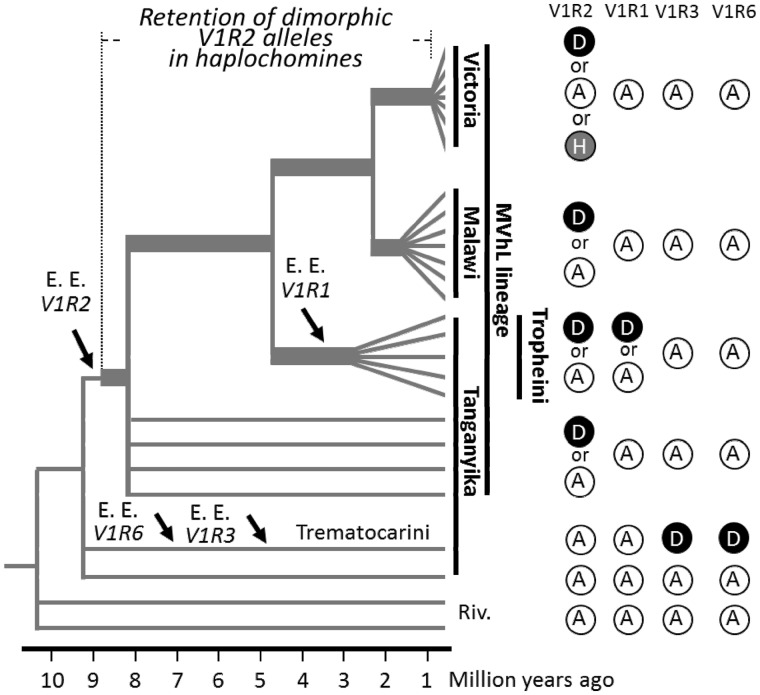
Multiple episodic evolution events and the diversity of V1Rs in cichlids. (*A*) The phylogenetic tree shown here is from a previous review (Kocher 2004). Arrows indicate the timing of episodic evolution (E. E., namely, positive selection) of each V1R gene. Gray radiating lines indicate the radiation events in LV, LM, and the tribe Tropheini. D and A indicate the species with derived and ancestral allele groups, respectively. H indicates the species, which is polymorphic with derived and ancestral alleles. Note that *V1R1* and *V1R2* are highly dimorphic in terms of ancestral and derived alleles. Furthermore, the highly dimorphic *V1R2* alleles were retained for several million years.

In addition to the signature of positive selection, it is of primary interest that highly dimorphic alleles of *V1R1* and *V1R2* appear to have been retained in the ancestral lineages. In particular, polymorphism of *V1R2* appears to have been retained for several million years (fig. 6). This phenomenon is illustrated by the incongruence between *V1R2* gene tree (fig. 3) and species tree of cichlids. Recent studies suggested that such incongruence between gene tree(s) and species tree is possibly caused by a demographic event such as hybridization of heterogeneous populations (Seehausen et al. 2003; Seehausen 2004) and/or retention of the ancestral polymorphisms (Loh et al. 2012). Importantly, such incongruence is expected to become more evident in genes under natural selection (Seehausen 2004). We speculate that the sharing of the two *V1R2* allele groups among cichlids of all three East-African lakes could be explained by this hypothesis. Similarly, retention of polymorphisms in several LV cichlid species could also be explained by hybridization and/or ancestral polymorphism, because these species (or populations) are quite young (Kocher 2004). Actually, a certain level of introgressions and the persistence of unfixed ancestral polymorphisms have been described in this group (Nagl et al. 1998; Samonte et al. 2007). More extensive evolutionary analysis using the draft genome sequences may provide insight into how highly dimorphic alleles of *V1R1* and *V1R2* emerged in the ancestral lineages and were retained during evolution.

### Evolution and Future Perspective on V1R-Mediated Social Communication in Cichlids

The phylogenetic tree of East-African cichlids depicts the episodic evolution and the resultant diversity of V1Rs (fig. 6). In this study, the signatures of episodic acceleration of amino acid substitutions were detected at least four times. Thus, the East-African cichlids possess several varieties of V1R combinations, in terms of ancestral and derived allele groups, which are expected to be functionally distinct in ligand recognition. Given that the repertoire and also the sequences of orthologous *V1R* genes are highly conserved among the distantly related fish species investigated (see Introduction), our finding of the high degree of sequence diversity in *V1R* genes among closely related cichlids is quite surprising. At present, V1Rs are expected to receive steroids or prostaglandins called “hormonal pheromones,” which ensure the synchronization of spawning interactions (Saraiva and Korsching 2007; Johansson and Banks 2010). Furthermore, Cole and Stacey (2006) demonstrated that *H. burtoni* can detect a variety of conjugated steroids, which increase serum testosterone in males. These findings imply that East-African cichlids may use V1Rs to detect such conjugated steroids during their reproductive interactions. Hence, our finding of the unexpected diversity of V1Rs may provide insights into the diversification of olfactory interactions in cichlids. Namely, in the case that each of two different V1R allele groups is fixed in two populations, it is possible that each of the population is distinct in detecting the hormonal pheromone(s) that may lead to the incipient step of population divergence because of the desynchronization of the spawning interactions between them. Specification of the ligands of each V1R and evaluation of the relevant effects in closely related cichlids possessing different *V1R* allele groups might elucidate the evolutionary biology of speciation.

## Supplementary Material

Supplementary tables S1 and S2 and figures S1–S6 are available at *Genome Biology and Evolution* online (http://www.gbe.oxfordjournals.org/).

Supplementary Data
