# Supplementary material for: Multiple Episodic Evolution Events in V1R Receptor Genes of East-African Cichlids
Source: Genome Biol Evol. 2014 May 6;6(5):1135–44. doi: 10.1093/gbe/evu086 (PMC4040994; doi:10.1093/gbe/evu086)
Supplement: Supplementary Data [file supp_6_5_1135__index.html]

Multiple episodic evolution events in V1R receptor genes of East-African cichlids — Multiple Episodic Evolution Events in V1R Receptor Genes of East-African Cichlids — Supplementary Data 

# Multiple Episodic Evolution Events in V1R Receptor Genes of East-African Cichlids

## Supplementary Data

files

**Files in this Data Supplement:**

- Supplementary Data - xls file
- Supplementary Data - pptx file
