# Supplementary material for: Multiple Episodic Evolution Events in V1R Receptor Genes of East-African Cichlids
Source: Genome Biol Evol. 2014 May 6;6(5):1135–44. doi: 10.1093/gbe/evu086 (PMC4040994; doi:10.1093/gbe/evu086)
Supplement: Supplementary Data [file supp_evu086_Supplementary_materials.pptx]

## Slide 1
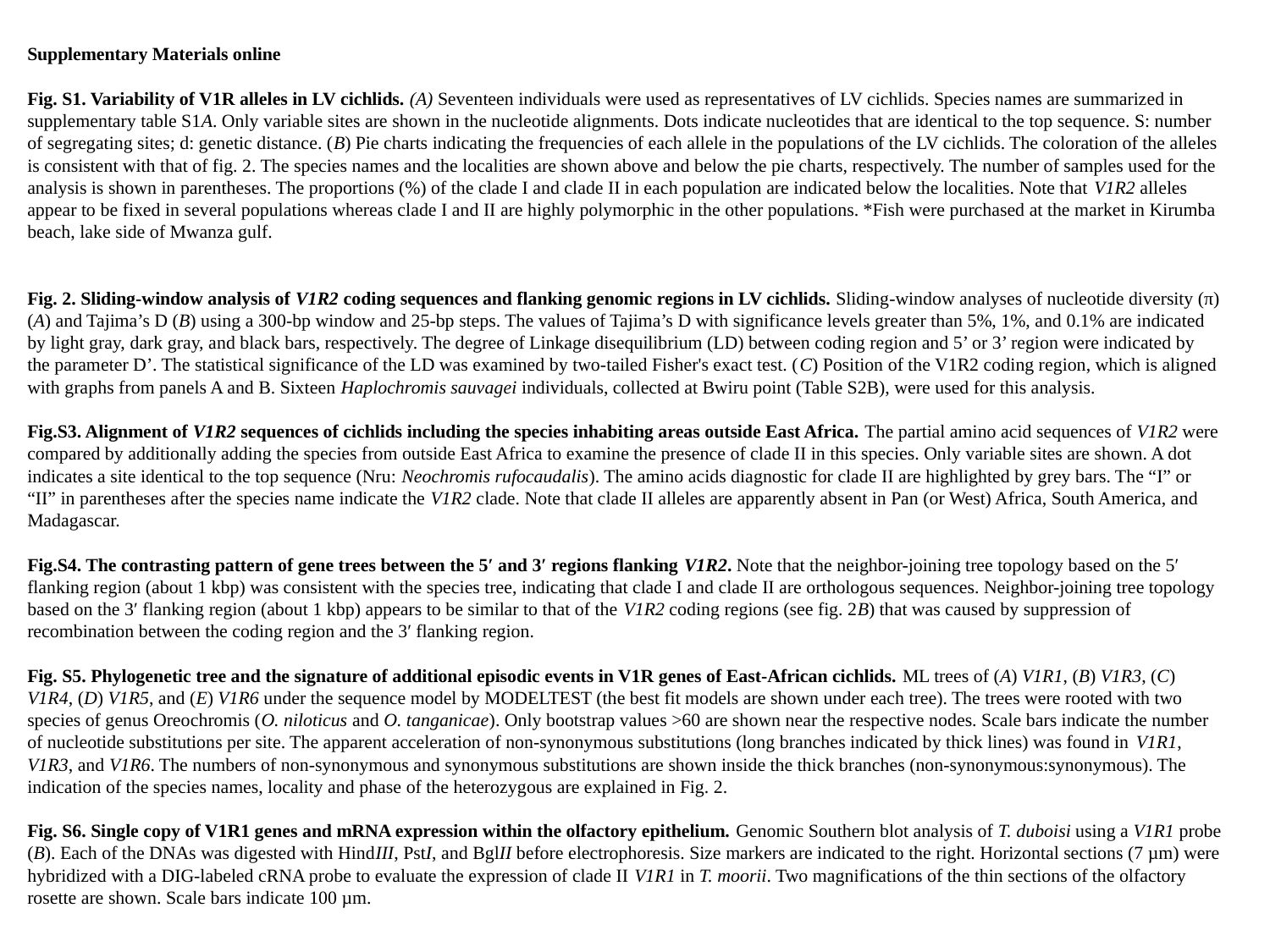

Supplementary Materials online
Fig. S1. Variability of V1R alleles in LV cichlids. (A) Seventeen individuals were used as representatives of LV cichlids. Species names are summarized in supplementary table S1A. Only variable sites are shown in the nucleotide alignments. Dots indicate nucleotides that are identical to the top sequence. S: number of segregating sites; d: genetic distance. (B) Pie charts indicating the frequencies of each allele in the populations of the LV cichlids. The coloration of the alleles is consistent with that of fig. 2. The species names and the localities are shown above and below the pie charts, respectively. The number of samples used for the analysis is shown in parentheses. The proportions (%) of the clade I and clade II in each population are indicated below the localities. Note that V1R2 alleles appear to be fixed in several populations whereas clade I and II are highly polymorphic in the other populations. *Fish were purchased at the market in Kirumba beach, lake side of Mwanza gulf.
Fig. 2. Sliding-window analysis of V1R2 coding sequences and flanking genomic regions in LV cichlids. Sliding-window analyses of nucleotide diversity (π) (A) and Tajima’s D (B) using a 300-bp window and 25-bp steps. The values of Tajima’s D with significance levels greater than 5%, 1%, and 0.1% are indicated by light gray, dark gray, and black bars, respectively. The degree of Linkage disequilibrium (LD) between coding region and 5’ or 3’ region were indicated by the parameter D’. The statistical significance of the LD was examined by two-tailed Fisher's exact test. (C) Position of the V1R2 coding region, which is aligned with graphs from panels A and B. Sixteen Haplochromis sauvagei individuals, collected at Bwiru point (Table S2B), were used for this analysis.
Fig.S3. Alignment of V1R2 sequences of cichlids including the species inhabiting areas outside East Africa. The partial amino acid sequences of V1R2 were compared by additionally adding the species from outside East Africa to examine the presence of clade II in this species. Only variable sites are shown. A dot indicates a site identical to the top sequence (Nru: Neochromis rufocaudalis). The amino acids diagnostic for clade II are highlighted by grey bars. The “I” or “II” in parentheses after the species name indicate the V1R2 clade. Note that clade II alleles are apparently absent in Pan (or West) Africa, South America, and Madagascar.
Fig.S4. The contrasting pattern of gene trees between the 5ʹ and 3ʹ regions flanking V1R2. Note that the neighbor-joining tree topology based on the 5ʹ flanking region (about 1 kbp) was consistent with the species tree, indicating that clade I and clade II are orthologous sequences. Neighbor-joining tree topology based on the 3ʹ flanking region (about 1 kbp) appears to be similar to that of the V1R2 coding regions (see fig. 2B) that was caused by suppression of recombination between the coding region and the 3ʹ flanking region.
Fig. S5. Phylogenetic tree and the signature of additional episodic events in V1R genes of East-African cichlids. ML trees of (A) V1R1, (B) V1R3, (C) V1R4, (D) V1R5, and (E) V1R6 under the sequence model by MODELTEST (the best fit models are shown under each tree). The trees were rooted with two species of genus Oreochromis (O. niloticus and O. tanganicae). Only bootstrap values >60 are shown near the respective nodes. Scale bars indicate the number of nucleotide substitutions per site. The apparent acceleration of non-synonymous substitutions (long branches indicated by thick lines) was found in V1R1, V1R3, and V1R6. The numbers of non-synonymous and synonymous substitutions are shown inside the thick branches (non-synonymous:synonymous). The indication of the species names, locality and phase of the heterozygous are explained in Fig. 2.
Fig. S6. Single copy of V1R1 genes and mRNA expression within the olfactory epithelium. Genomic Southern blot analysis of T. duboisi using a V1R1 probe (B). Each of the DNAs was digested with HindIII, PstI, and BglII before electrophoresis. Size markers are indicated to the right. Horizontal sections (7 µm) were hybridized with a DIG-labeled cRNA probe to evaluate the expression of clade II V1R1 in T. moorii. Two magnifications of the thin sections of the olfactory rosette are shown. Scale bars indicate 100 µm.

## Slide 2
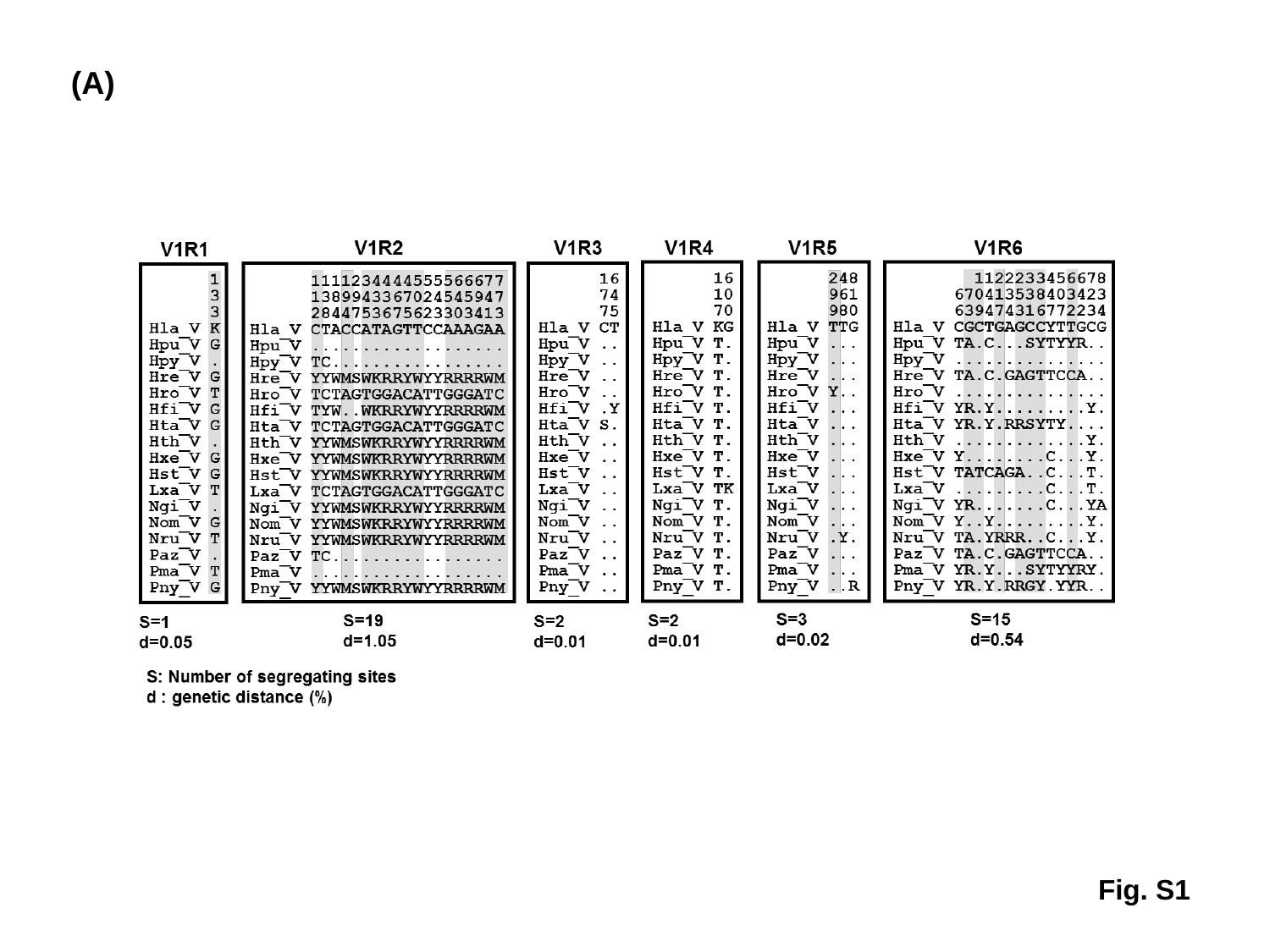

(A)
Fig. S1

## Slide 3
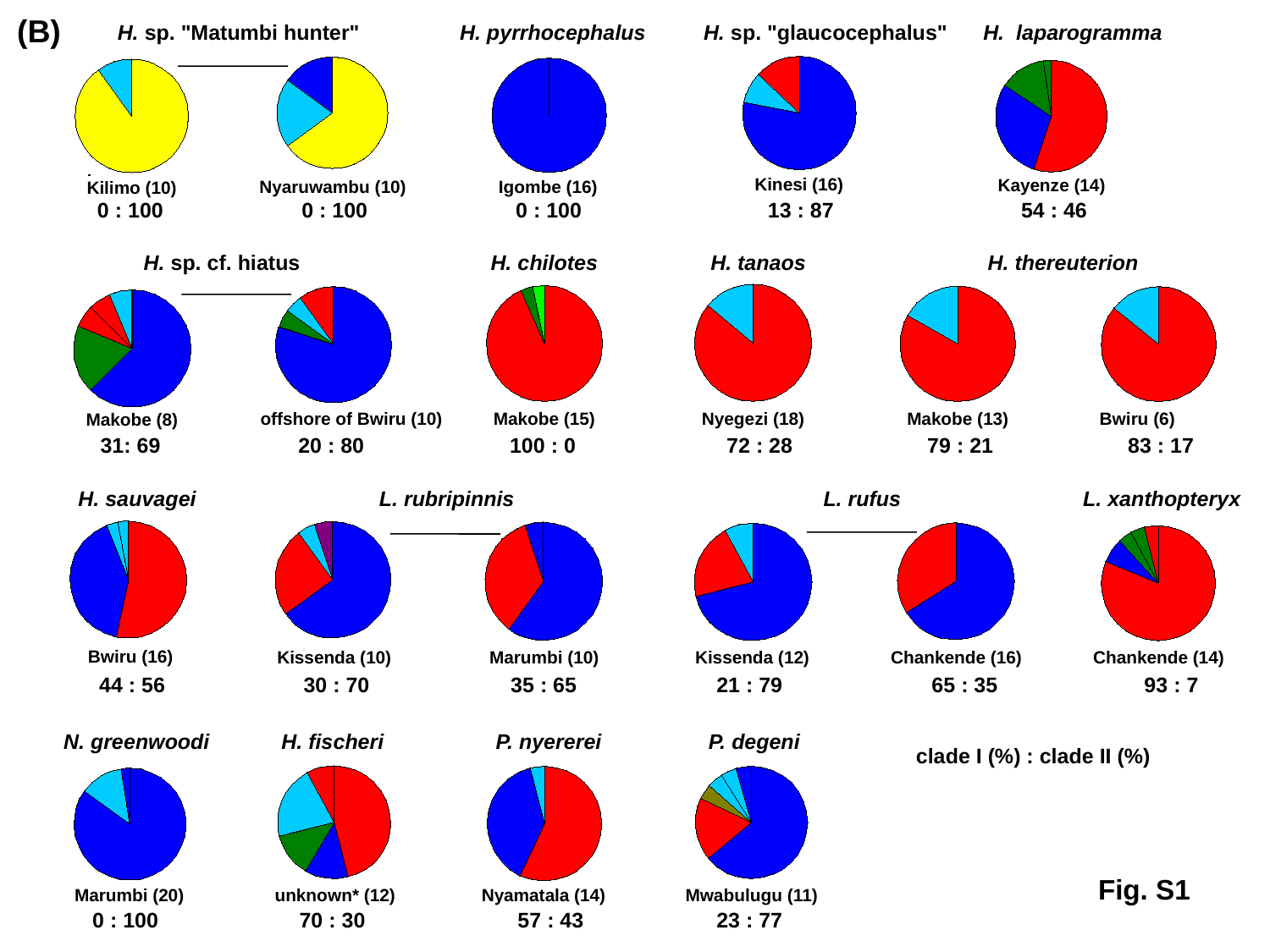

(B)
H. sp. "Matumbi hunter"
H. pyrrhocephalus
H. sp. "glaucocephalus"
H. laparogramma
Kinesi (16)
Nyaruwambu (10)
Igombe (16)
Kilimo (10)
Kayenze (14)
0 : 100
0 : 100
0 : 100
13 : 87
54 : 46
H. sp. cf. hiatus
H. chilotes
H. tanaos
H. thereuterion
Nyegezi (18)
Makobe (15)
Makobe (13)
offshore of Bwiru (10)
Bwiru (6)
Makobe (8)
31: 69
20 : 80
100 : 0
72 : 28
79 : 21
83 : 17
H. sauvagei
L. rubripinnis
L. rufus
L. xanthopteryx
Bwiru (16)
Kissenda (10)
Marumbi (10)
Chankende (16)
Kissenda (12)
Chankende (14)
44 : 56
30 : 70
35 : 65
21 : 79
65 : 35
93 : 7
N. greenwoodi
H. fischeri
P. nyererei
P. degeni
clade I (%) : clade II (%)
unknown* (12)
Nyamatala (14)
Mwabulugu (11)
Marumbi (20)
Fig. S1
0 : 100
70 : 30
57 : 43
23 : 77

## Slide 4
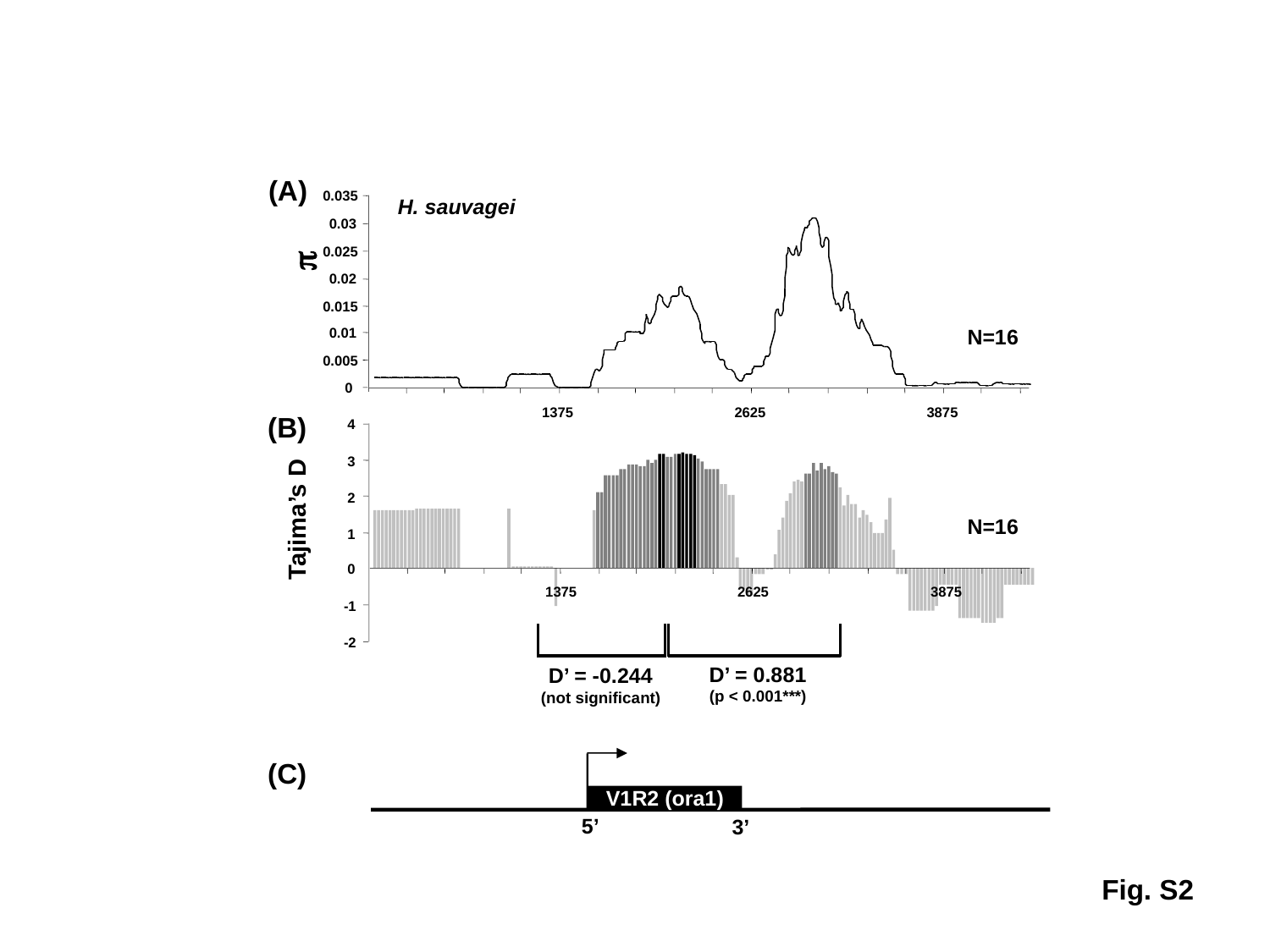

(A)
0.035
H. sauvagei
0.03
p
0.025
0.02
0.015
N=16
0.01
0.005
0
(B)
1375
2625
3875
4
3
2
Tajima’s D
N=16
1
0
1375
2625
3875
-1
-2
D’ = 0.881
(p < 0.001***)
D’ = -0.244
(not significant)
(C)
V1R2 (ora1)
5’
3’
Fig. S2

## Slide 5
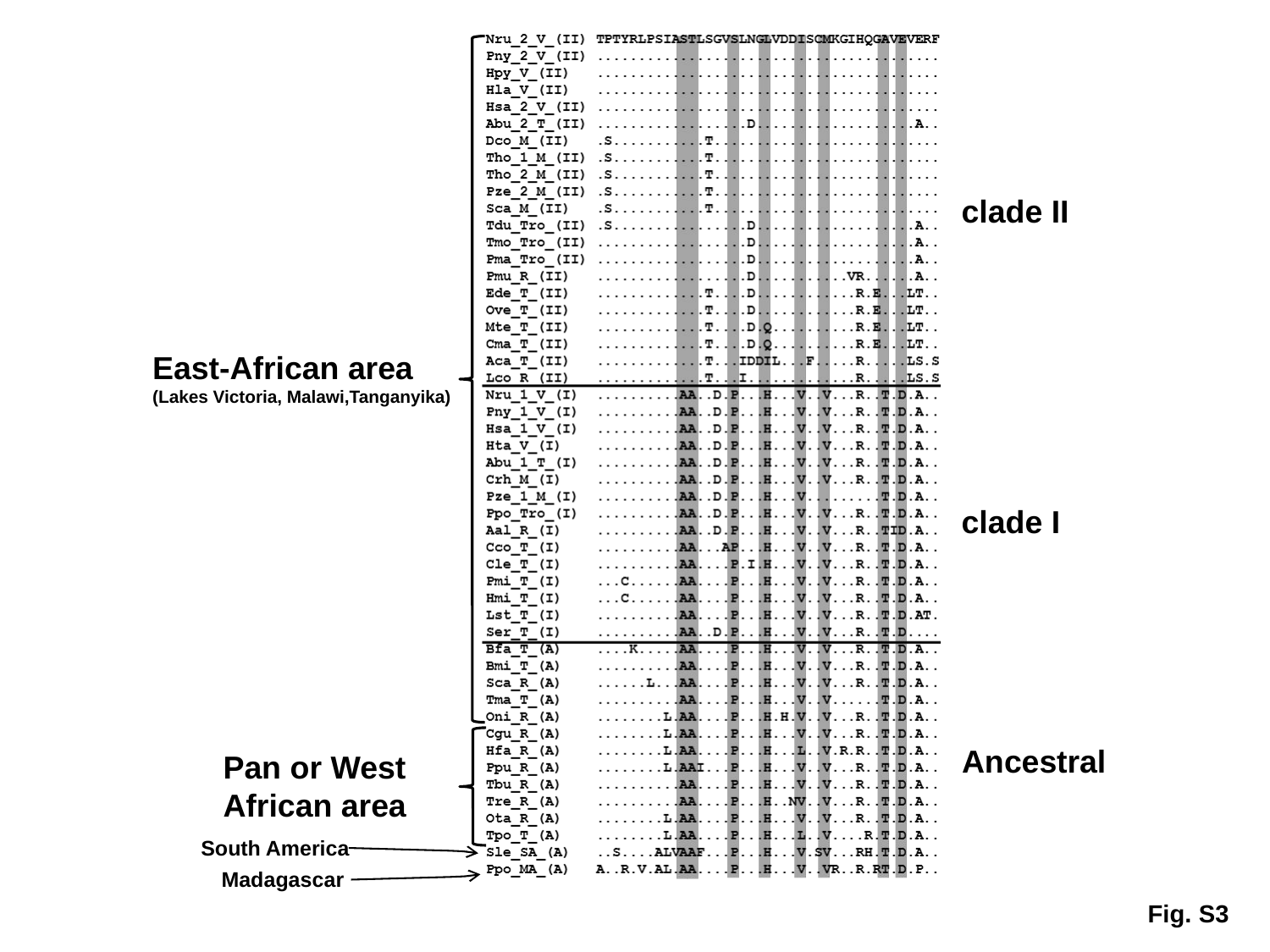

clade II
East-African area
(Lakes Victoria, Malawi,Tanganyika)
clade I
Ancestral
Pan or West
African area
South America
Madagascar
Fig. S3

## Slide 6
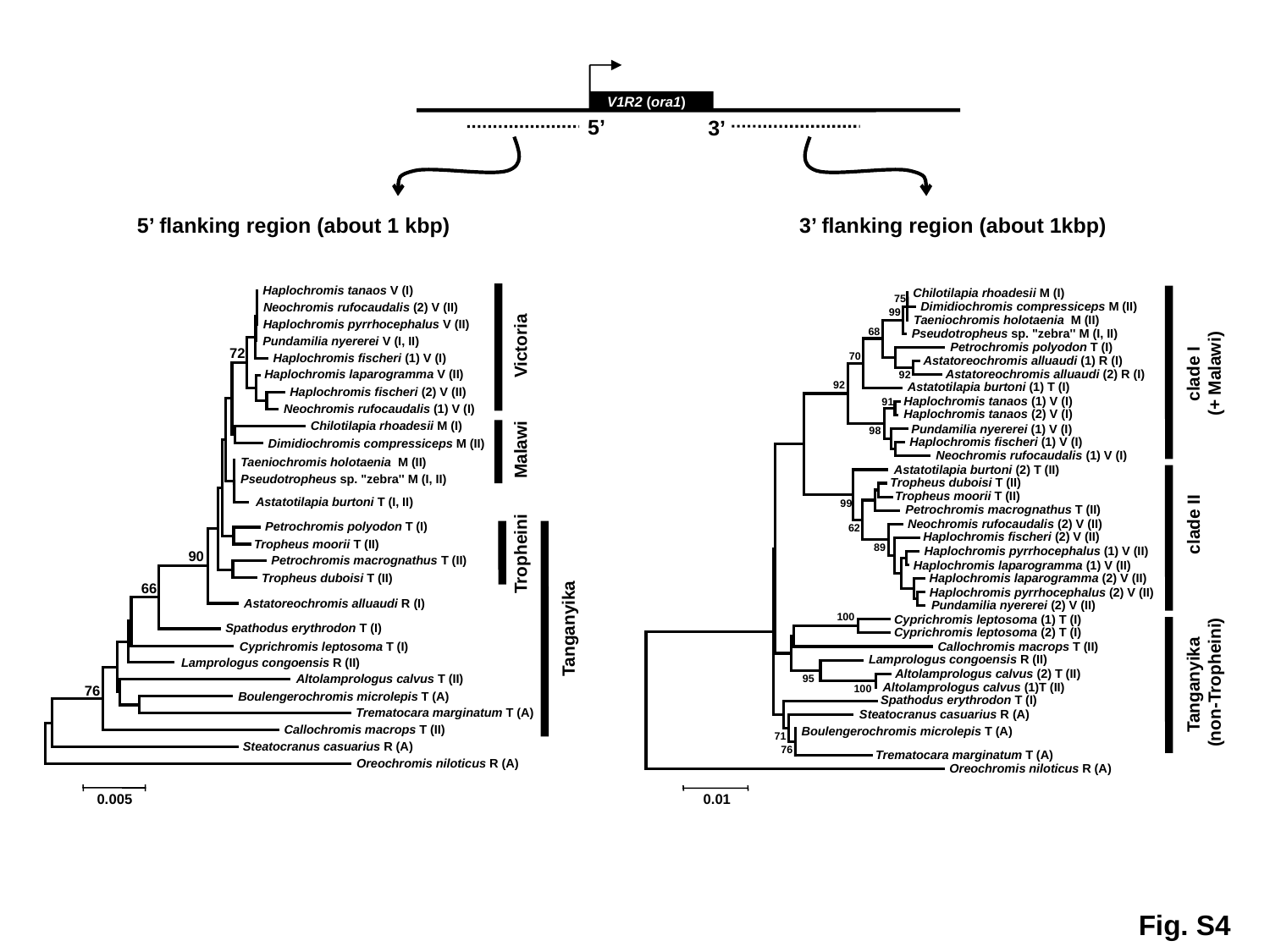

V1R2 (ora1)
5’
3’
5’ flanking region (about 1 kbp)
3’ flanking region (about 1kbp)
 Haplochromis tanaos V (I)
 Neochromis rufocaudalis (2) V (II)
 Haplochromis pyrrhocephalus V (II)
Victoria
 Pundamilia nyererei V (I, II)
72
 Haplochromis fischeri (1) V (I)
Haplochromis laparogramma V (II)
 Haplochromis fischeri (2) V (II)
 Neochromis rufocaudalis (1) V (I)
 Chilotilapia rhoadesii M (I)
Malawi
 Dimidiochromis compressiceps M (II)
 Taeniochromis holotaenia M (II)
 Pseudotropheus sp. "zebra'' M (I, II)
 Astatotilapia burtoni T (I, II)
 Petrochromis polyodon T (I)
Tropheus moorii T (II)
Tropheini
90
 Petrochromis macrognathus T (II)
 Tropheus duboisi T (II)
66
Astatoreochromis alluaudi R (I)
Tanganyika
 Spathodus erythrodon T (I)
 Cyprichromis leptosoma T (I)
 Lamprologus congoensis R (II)
 Altolamprologus calvus T (II)
76
Boulengerochromis microlepis T (A)
 Trematocara marginatum T (A)
 Callochromis macrops T (II)
Steatocranus casuarius R (A)
Oreochromis niloticus R (A)
0.005
 Chilotilapia rhoadesii M (I)
75
 Dimidiochromis compressiceps M (II)
99
 Taeniochromis holotaenia M (II)
68
 Pseudotropheus sp. "zebra'' M (I, II)
 Petrochromis polyodon T (I)
clade I
(+ Malawi)
70
Astatoreochromis alluaudi (1) R (I)
Astatoreochromis alluaudi (2) R (I)
92
92
 Astatotilapia burtoni (1) T (I)
 Haplochromis tanaos (1) V (I)
91
 Haplochromis tanaos (2) V (I)
 Pundamilia nyererei (1) V (I)
98
 Haplochromis fischeri (1) V (I)
 Neochromis rufocaudalis (1) V (I)
 Astatotilapia burtoni (2) T (II)
 Tropheus duboisi T (II)
Tropheus moorii T (II)
99
 Petrochromis macrognathus T (II)
clade II
 Neochromis rufocaudalis (2) V (II)
62
 Haplochromis fischeri (2) V (II)
89
 Haplochromis pyrrhocephalus (1) V (II)
Haplochromis laparogramma (1) V (II)
Haplochromis laparogramma (2) V (II)
 Haplochromis pyrrhocephalus (2) V (II)
 Pundamilia nyererei (2) V (II)
100
 Cyprichromis leptosoma (1) T (I)
 Cyprichromis leptosoma (2) T (I)
 Callochromis macrops T (II)
 Lamprologus congoensis R (II)
Tanganyika
(non-Tropheini)
 Altolamprologus calvus (2) T (II)
95
 Altolamprologus calvus (1)T (II)
100
 Spathodus erythrodon T (I)
Steatocranus casuarius R (A)
Boulengerochromis microlepis T (A)
71
76
 Trematocara marginatum T (A)
Oreochromis niloticus R (A)
0.01
Fig. S4

## Slide 7
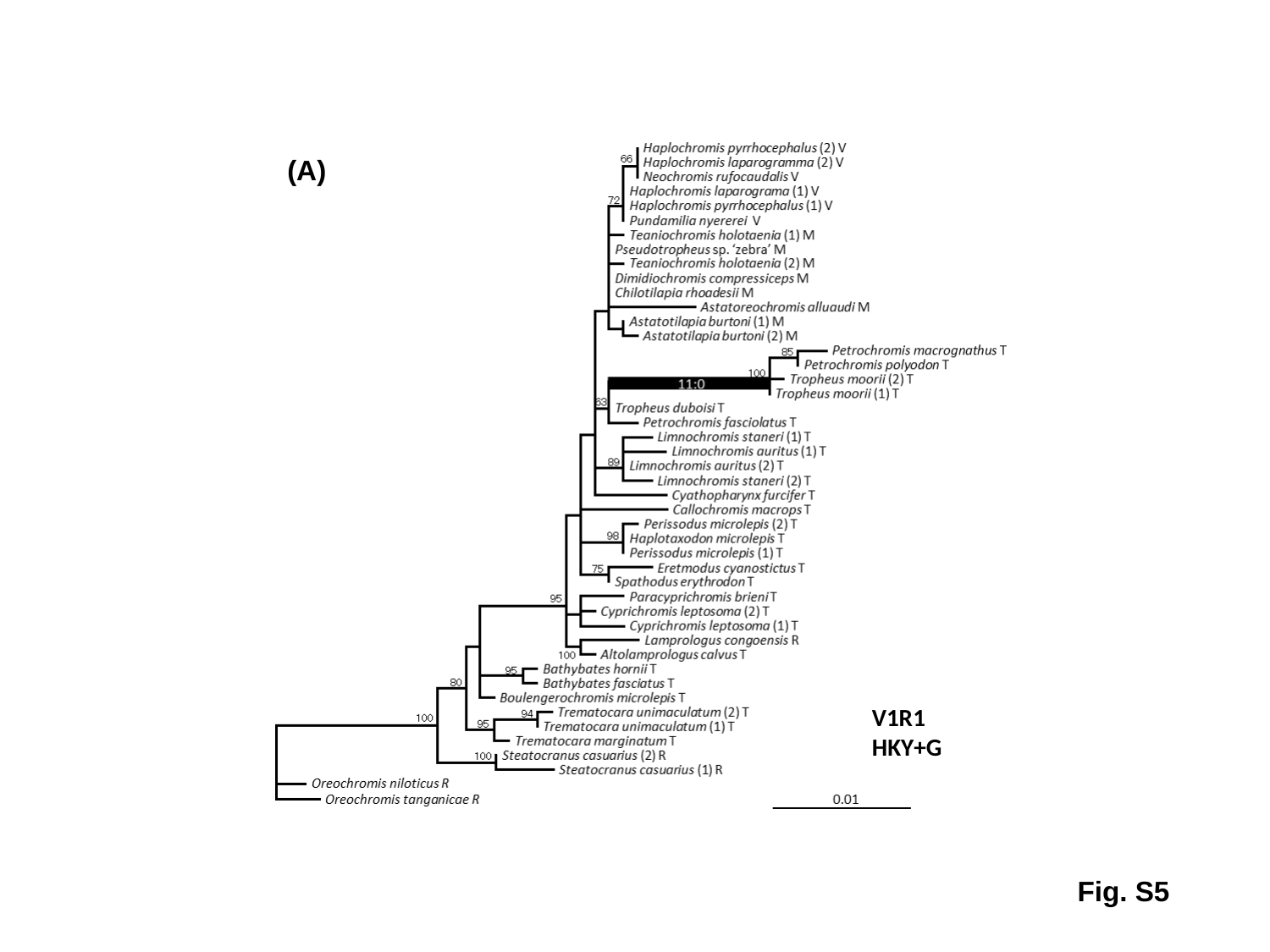

(A)
V1R1
HKY+G
Fig. S5

## Slide 8
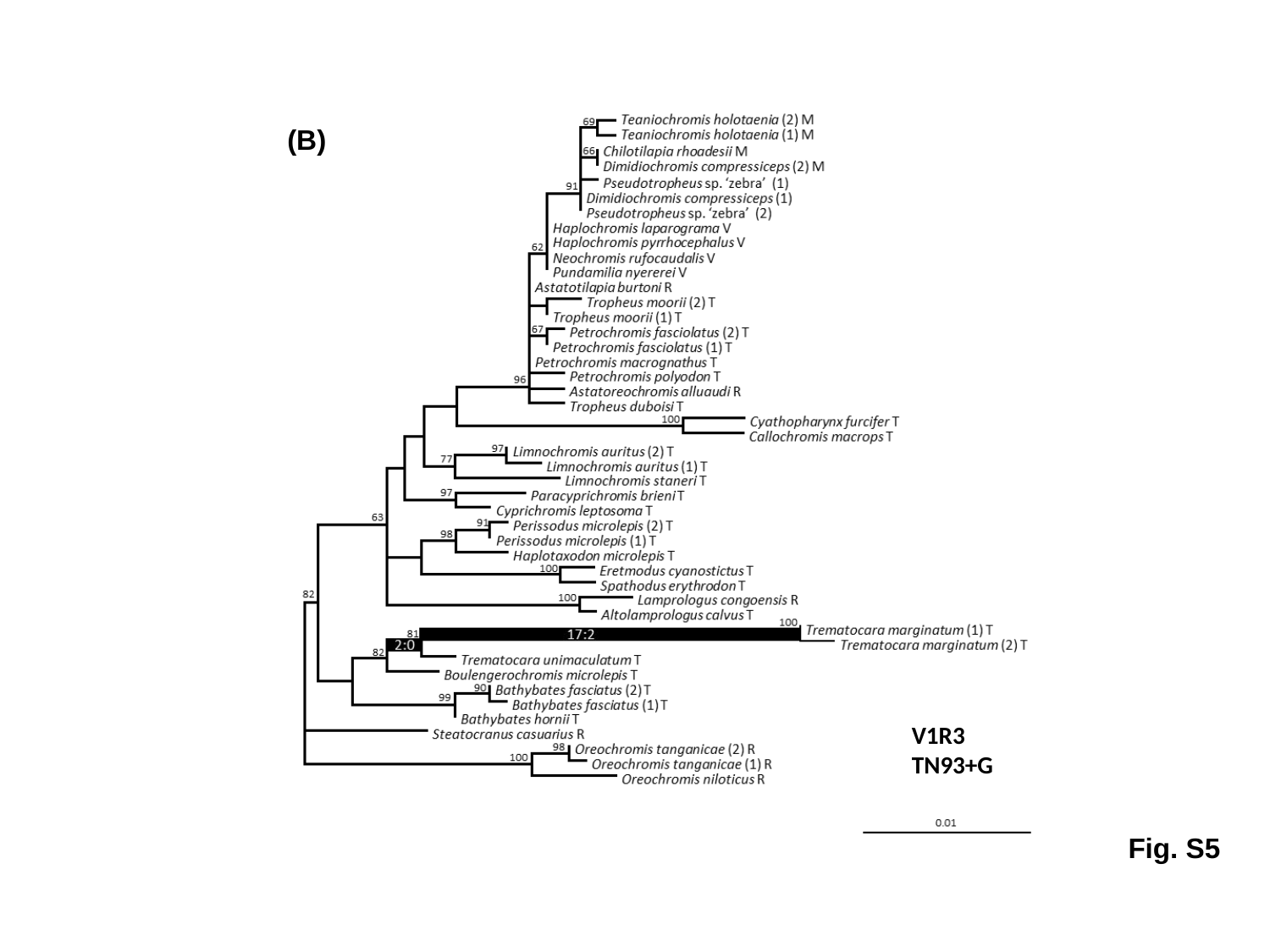

(B)
V1R3
TN93+G
Fig. S5

## Slide 9
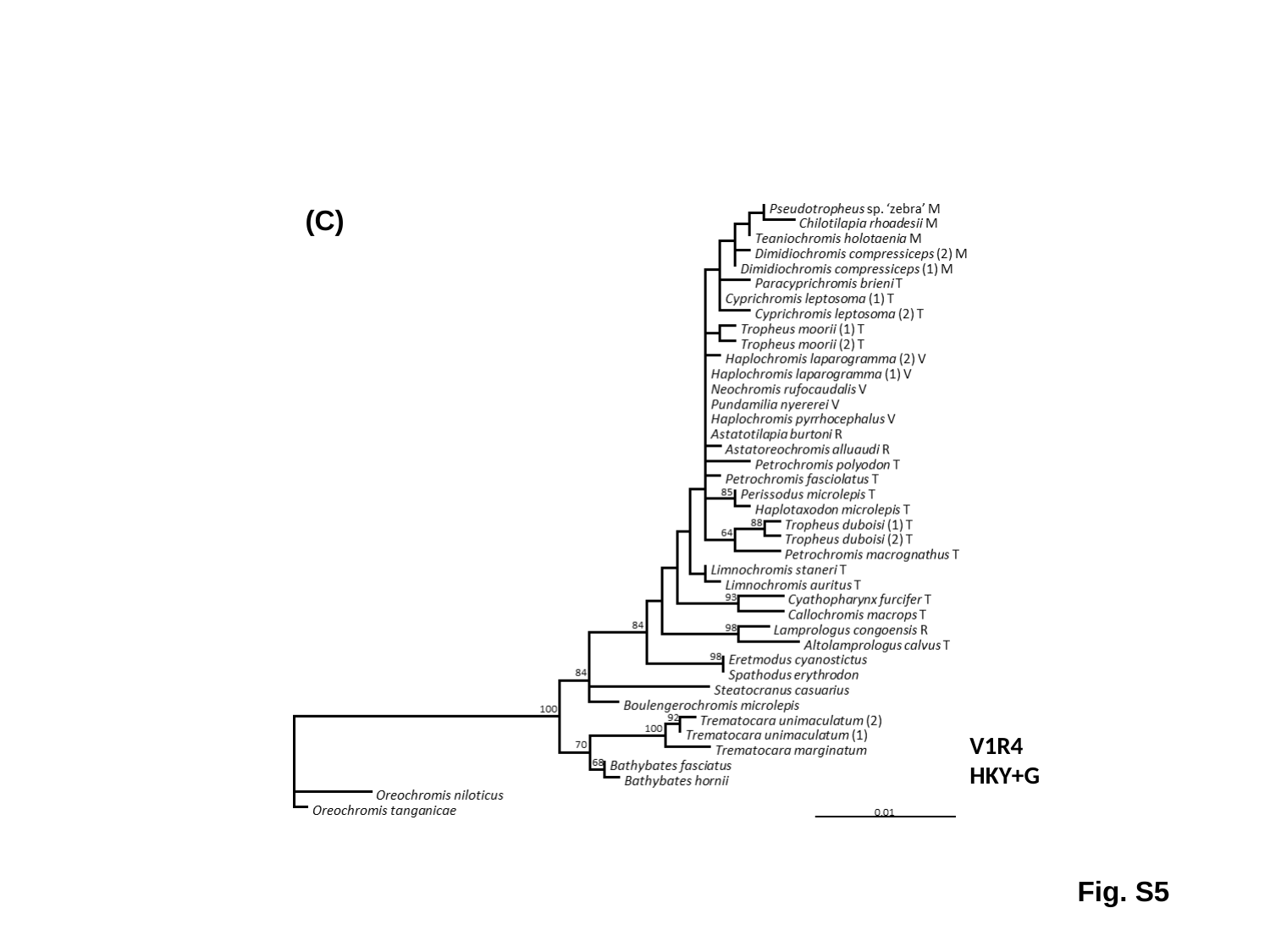

(C)
V1R4
HKY+G
Fig. S5

## Slide 10
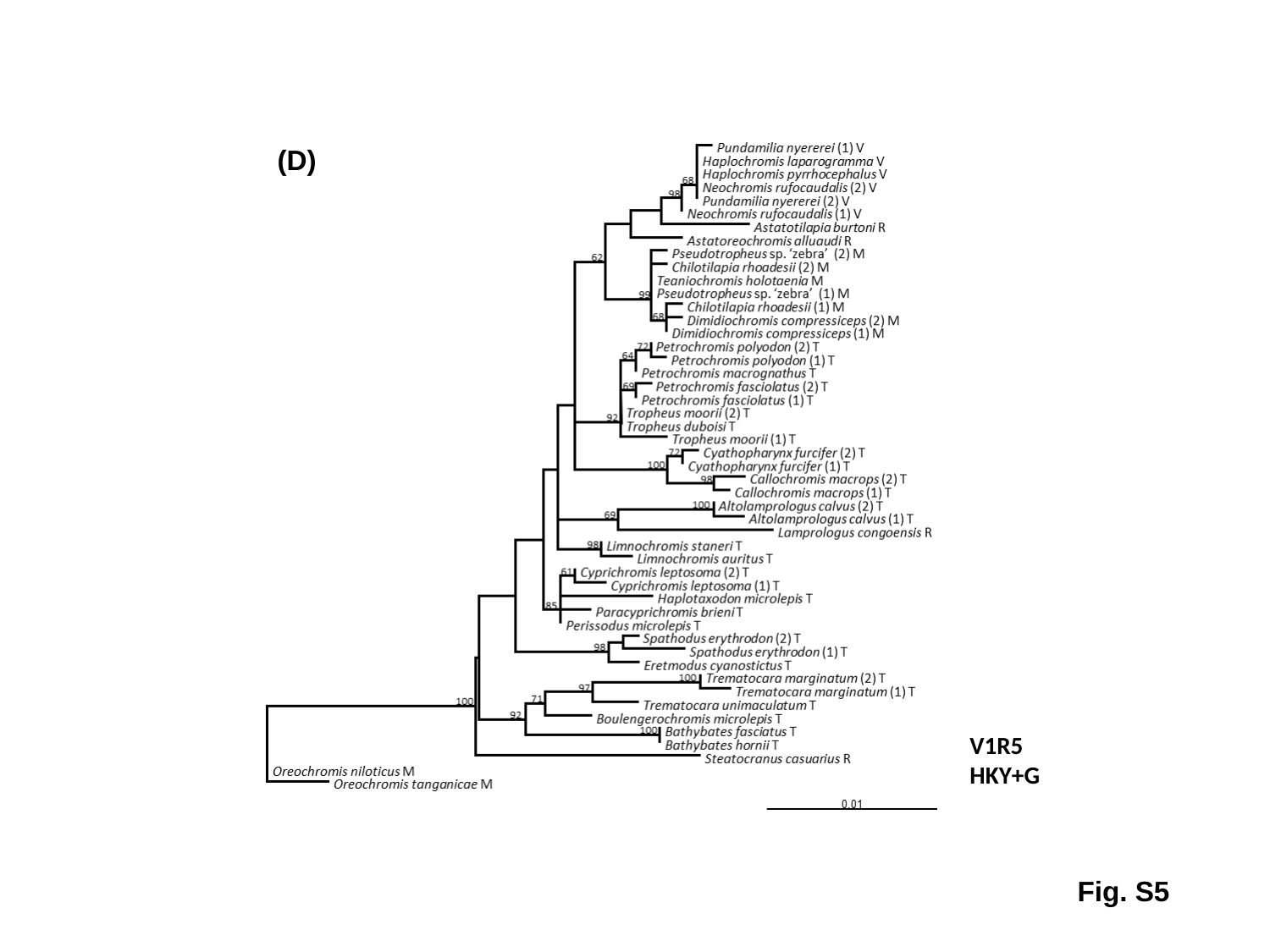

(D)
V1R5
HKY+G
Fig. S5

## Slide 11
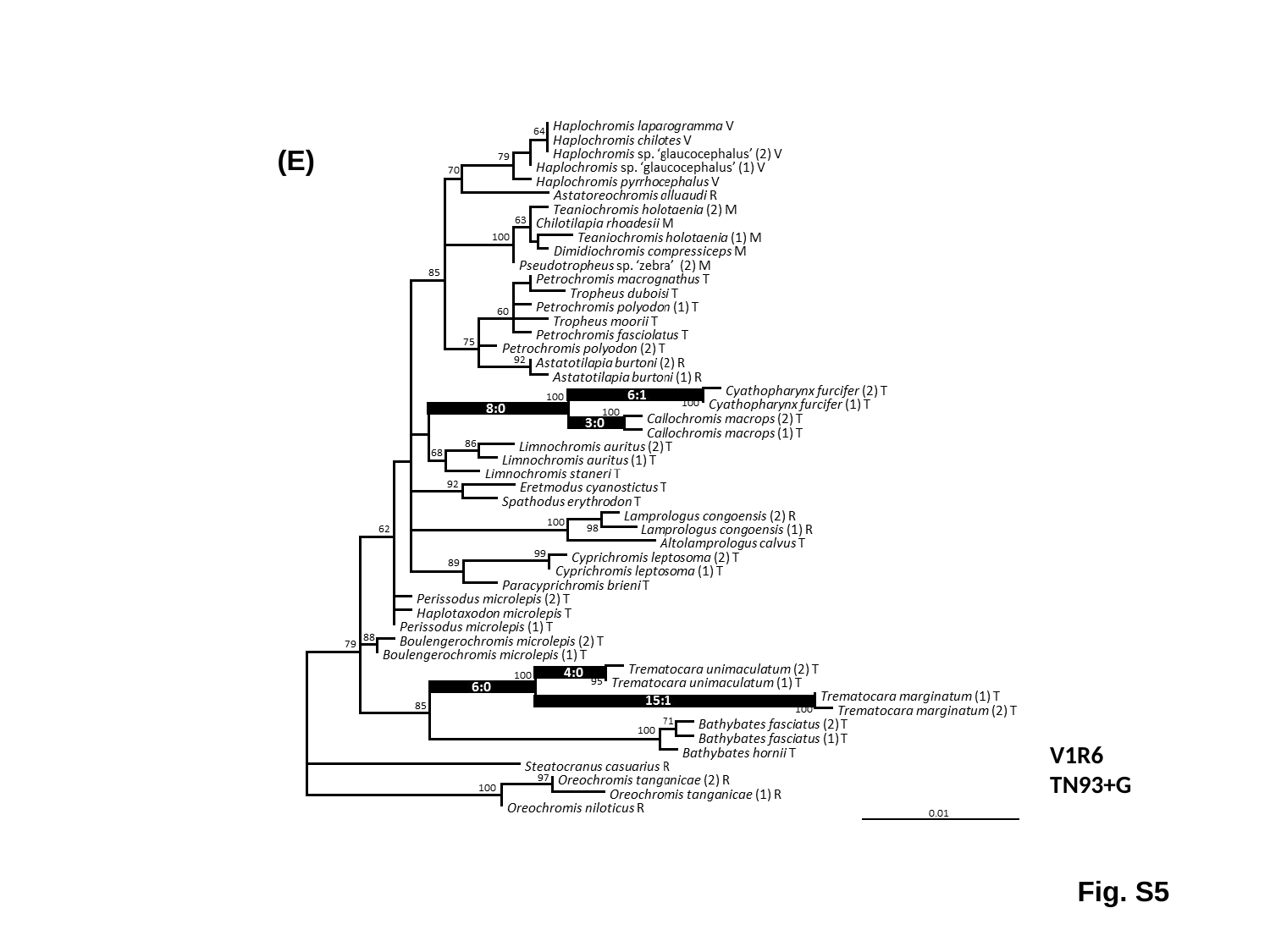

(E)
V1R6
TN93+G
Fig. S5

## Slide 12
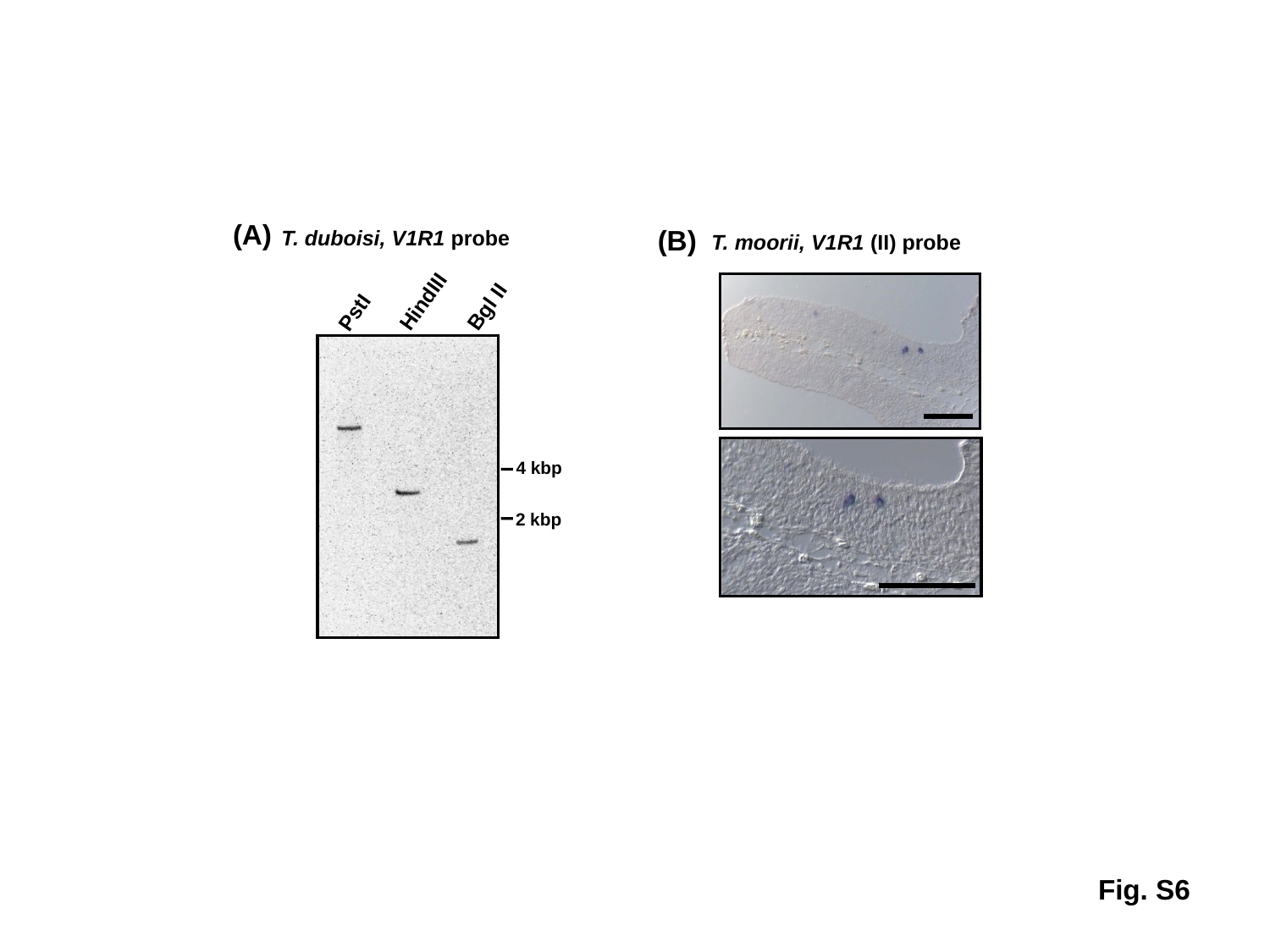

(A)
T. duboisi, V1R1 probe
(B)
T. moorii, V1R1 (II) probe
HindIII
Bgl II
PstI
4 kbp
2 kbp
Fig. S6
